# TED—trazodone effectiveness in depression: a naturalistic study of the effeciveness of trazodone in extended release formulation compared to SSRIs in patients with a major depressive disorder

**DOI:** 10.3389/fphar.2023.1296639

**Published:** 2023-11-01

**Authors:** Dominika Dudek, Adrian Andrzej Chrobak, Anna Julia Krupa, Aleksandra Gorostowicz, Adrian Gerlich, Andrzej Juryk, Marcin Siwek

**Affiliations:** ^1^ Department of Adult Psychiatry, Jagiellonian University Medical College, Cracow, Poland; ^2^ Department of Affective Disorders, Jagiellonian University Medical College, Cracow, Poland; ^3^ Department of Adult, Child and Adolescent Psychiatry, University Hospital in Cracow, Cracow, Poland

**Keywords:** depression, major depressive disorder, trazodone, selective serotonin reuptake inhibitor, insomnia, anhedonia, anxiety

## Abstract

**Introduction:** Selective serotonin reuptake inhibitors (SSRIs) are the most often used medications to treat major depressive disorder (MDD). Despite their effectiveness in reducing depressive symptoms, several issues are associated with their use in MDD, such as limited improvement of anhedonia, emergence of emotional blunting, induction or exacerbation of insomnia, and sexual dysfunction. Due to its also devoid of the issues related to treatment noted with SSRIs. The aim of this 12-week non-inferiority naturalistic observation was to compare the effectiveness and tolerability of SSRIs and trazodone in extended release (XR) in MDD.

**Methods:** A total of 186 subjects were recruited, of which 92 received trazodone XR and 94 received SSRIs. Patients were allocated to trazodone XR or SSRIs, according to the attending physician based on clinical evaluation. Assessments at baseline and weeks 2, 4, 8, and 12 were conducted to evaluate the severity of depression (Montgomery–Åsberg Depression Rating Scale, clinician- and patient-rated Quick Inventory of Depressive Symptomatology—the primary endpoints of the study), anhedonia (the Snaith–Hamilton Pleasure Scale), anxiety (the Hamilton Anxiety Rating Scale), insomnia (the Athens Insomnia Scale), and therapeutic effectiveness (the Clinical Global Impression Scale).

**Results:** After 12 weeks, trazodone XR was more effective than SSRIs in reducing the severity of depression, anxiety, and insomnia. There was a trend for higher effectiveness of in reduction of anhedonia, which became insignificant after controlling the results for the duration of previous psychiatric treatment as a covariate. The proportion of treatment-responsive subjects in the trazodone XR group compared to SSRIs was comparable or higher. The proportion of patients achieving remission was higher in the trazodone XR arm vs. the SSRI arm.

**Discussion:** In summary, the results indicate that trazodone XR is effective in MDD in the “real-world” setting. Its potential superiority over SSRIs in addressing particular symptomatic dimensions should be verified in future studies.

## 1 Introduction

It is estimated that depression affects 10% of the general population (Qaseem et al., 2023). According to the Global Burden of Disease Study 2017, depression is the third leading cause of the burden of years lived with disability (James et al., 2018). Therefore, the optimization of depression treatment is a public health priority. The perspectives on the goals of depression treatment shift depending on its phase. Although, in the acute phase, the primary objective of the therapy is to reduce the symptoms, ideally to the level of remission, the maintenance is aimed at sustaining remission and preventing relapse. Finally, recovery is intended to restore the patient to the previous level of functioning (Qaseem et al., 2023).

As shown by Cipriani et al., there are many molecules which are effective in the treatment of major depressive disorder (MDD) (Cipriani et al., 2018). Currently, selective serotonin reuptake inhibitors (SSRIs) are the most often used and are commonly suggested as the first-line MDD treatment (Latendresse et al., 2017; APA. American Psychological Association, 2019; Marasine et al., 2021; NICE. National Institute for Health and Care Excellence, 2022; Qaseem et al., 2023). Although SSRIs are generally effective in reducing the symptoms of depression, several problems are linked to their use. Recent studies indicated that SSRIs might not only be ineffective in decreasing anhedonia (Yee et al., 2015) but they might also cause emotional blunting in as much as 50%–60% of patients (Price et al., 2009; Goodwin et al., 2017). Given that anhedonia and emotional blunting mediate the improvement of overall depression symptoms, general functioning, and quality of life, their persistence and exacerbation might hinder the achievement of recovery (McMakin et al., 2012; Siwek, 2017; Fagiolini et al., 2021). Furthermore, in some patients, SSRIs do not improve and might even worsen insomnia, which is one of the main causes of non-adherence to SSRIs in MDD (Badamasi et al., 2019). The majority of clinicians manage insomnia in patients taking SSRIs with add-on trazodone (Dording et al., 2002). Another symptom dimension of MDD, which is often either unresponsive or aggravated by SSRIs, is sexual functioning. Drug-induced sexual dysfunction is among the most common reasons for patients choosing to withdraw SSRI medication (Atmaca, 2020). Again, in order to manage sexual dysfunction due to SSRIs, physicians most often opt for the addition of another drug, bupropion (Dording et al., 2002). Yet, most depression treatment guidelines suggest antidepressant monotherapy as the preferred treatment option and rightly so as combined pharmacotherapy is linked to a higher risk of drug-to-drug interactions and adverse effects. Hence, while indisputably effective in MDD treatment, SSRIs might be inadequate or even disadvantageous in patients with pronounced anhedonia, insomnia, and sexual dysfunction or those in post-acute phases of MDD treatment.

Trazodone is an antidepressant medication classified as the serotonin 5-HT2 receptor antagonist and reuptake inhibitor (SARI). It is approved by the European Medicines Agency (Europe) and the Food and Drug Administration (United States of America) for the treatment of MDD in adult patients. Recommendations for trazodone use in clinical practice suggest that trazodone is helpful in MDD comorbid with anxiety, insomnia, and in MDD with psychomotor agitation (Cuomo et al., 2019; Albert et al., 2023; Fagiolini et al., 2023). A number of trials were conducted to compare trazodone vs. other antidepressants. Studies comparing trazodone vs. tricyclic antidepressants (TCA) in MDD treatment indicated that trazodone was comparable or superior vs. TCA; some suggested higher tolerability of trazodone (Fagiolini et al., 2012; Cuomo et al., 2019). More recent data showed that trazodone was as effective as SSRIs in MDD treatment, and more beneficial in patients with marked insomnia and was quicker to improve sleep quality (Papakostas and Fava, 2007). Trazodone was also less likely to induce sexual dysfunction than SSRIs (Khazaie et al., 2015). The comparison of trazodone with venlafaxine revealed that trazodone was more advantageous in improving insomnia, while venlafaxine offered greater effects in alleviating retardation and cognitive dysfunction, which might be due to its dual mechanism of action (serotonin and norepinephrine reuptake inhibition) (Cunningham et al., 1994; Czerwińska and Pawłowski, 2020). The majority of research on trazodone effectiveness in MDD was conducted in patients taking either immediate-release (IR) or continued-release (CR) formulation of this drug. However, in order to improve the compliance and tolerance of treatment, an extended-release formulation (also known as trazodone Contramid^®^ once-a day— COAD) was introduced. Compared to IR and CR formulations, XR presents a preferable pharmacokinetic profile. Due to the long half-life, the plasma concentration is characterized by a slower increase, lower peak, and gradual decrease. Given that the adverse effects are more likely to occur when the peak plasma concentrations of the drug are higher and change rapidly, trazodone XR might be better tolerated than IR or CR. Unlike trazodone IR or CR, XR is dosed once daily, which makes it easier for the patient to adhere to treatment (Fagiolini et al., 2012). Recently, two randomized clinical trials (RCTs) were performed to assess the effectiveness and tolerance of trazodone XR vs. other antidepressants. The first showed that while venlafaxine offered greater overall symptom reduction after 8 weeks of treatment, trazodone XR was more effective in achieving early response after 1 week of therapy (Fagiolini et al., 2020). The second indicated that trazodone XR was as effective as clomipramine but better tolerated (Buoli et al., 2019). Although RCTs are the “gold standard” of evidence-based assessments of drug effectiveness and tolerance, their results are not easily translated into every day clinical practice as due to strict inclusion criteria only 20% of patients are eligible (Preskorn et al., 2015). Thus, naturalistic observations are necessary to verify the effectiveness and tolerability of drugs in “real-world” patients. We have previously published the pilot study, which indicated that trazodone is not inferior to SSRIs in achieving the treatment response and remission in MDD patients (Siwek et al., 2023). Nonetheless, these results were preliminary and needed to be corroborated in a larger group of patients.

The aim of this study was to compare the effectiveness and tolerability of trazodone XR vs. SSRIs.

## 2 Materials and methods

### 2.1 Study design

This was a single-center, open-label, non-inferiority naturalistic observation comparing the effectiveness and tolerability of trazodone XR vs. SSRIs. For this study, patients with MDD diagnosed according to DSM-5 (Diagnostic and Statistical Manual of Mental Disorders 5th edition) criteria were included. The inclusion criteria are as follows: age 18–65 years, diagnosis of a first MDD episode according to DSM-5, or an acute MDD episode in patients diagnosed with recurrent depression. The exclusion criteria were as follows: current or a past episode of drug-resistant depression; diagnosis of bipolar disorder, persistent mood disorder, organic mood disorder, and schizoaffective disorder; substance use disorder (with the exception of nicotine and caffeine); pregnancy or breastfeeding; non-consensual treatment; severe somatic diseases associated with renal, hepatic, circulatory, or respiratory failure; a diagnosis of severe neurological disease (multiple sclerosis, neurodegenerative diseases, Parkinson’s disease, epilepsy, and dementia); and pharmacotherapy with clinically significant cytochrome P450 inducers, e.g., rifampicin, glucocorticosteroids, phenytoin, and carbamazepine.

Patients were allocated to groups receiving trazodone XR (150–300 mg/d) or SSRIs (sertraline 50–200 mg/d, citalopram 20–40 mg/d, escitalopram 10–20 mg/d, and paroxetine 20–60 mg/d) in monotherapy. The choice of the drug and its dose was made by the attending physician after thorough analysis of the clinical condition, potential comorbidities, and drug interactions. As in our pilot study, selection of the antidepressant was based on the clinical manifestation of MDD and previous treatment history, following the guidelines of the Polish Psychiatric Association and the National Consultant for Adult Psychiatry in Poland (Samochowiec et al., 2021; Siwek et al., 2023).

The study was approved by the Bioethics Committee of the Jagiellonian University in Krakow (approval No. 1072.6120.113.2021). All participants signed informed written consent forms.

### 2.2 Assessments

Basic socio-demographic data were collected by the attending physician at the enrollment. Clinical evaluation was performed at baseline and after 2, 4, 8, and 12 weeks of treatment. Each assessment included measures of overall depression severity, anxiety, anhedonia, and insomnia, which were conducted with the following:

• Depression rating scales: the Montgomery–Åsberg Depression Rating Scale (MADRS)—clinician-rated; the Quick Inventory of Depressive Symptomatology (QIDS)—clinician- (QIDS-CR) and self-rated (QIDS-SR),
• Clinician-rated tool measuring anxiety: the Hamilton Anxiety Rating Scale (HAM-A),
• Self-rated questionnaire to assess anhedonia: the Snaith–Hamilton Pleasure Scale (SHAPS),
• Self-rated scale to evaluate sleep disturbance: the Athens Insomnia Scale (AIS),
• Clinician-rated measure of overall symptom severity and improvement: the Clinical Global Impression Scale (CGI).

Changes in overall severity of depression measured by MADRS, QIDS-CR, and QIDS-SR scores across the subsequent time points were the primary endpoints of this study. The treatment response was defined as ≥50% reduction of symptoms, as assessed with QIDS, QIDS-SR, and MADRS or CGI-I score ≤2 (“Very Much Improved” or “Much Improved”), and the remission was defined as scores below 6 points on the QIDS-CR and QIDS-SR scale or below 10 on MADRS. Both treatment response and remission were assessed after 12 weeks of treatment duration.

### 2.3 Statistical analysis

Statistical analysis was carried out on data from 160 subjects who participated in the study. General group characteristics and baseline clinical measures were compared with the use of a *t*-test in the case of quantitative variables and χ2 in the case of qualitative variables between the groups receiving trazodone XR or SSRIs. The Shapiro–Wilk test was used to assess the distribution of quantitative variables. Qualitative variables were presented as proportions, and quantitative variables, as mean and standard deviations.

To evaluate the changes in the total scores of MADRS, QIDS-CR, and QIDS-SR (primary endpoints of the study), and HAM-A, AIS, and SHAPS (secondary endpoints), a linear mixed-effects model (MMRM — mixed model for repeated measures) was built. The analysis was performed via the lmer function from the lme4 package in R [version R 4.2.1 (RCore Team, 2022)]. The model included time points of measurement (0, 2, 4, 8, and 12 weeks) and the treatment group (trazodone XR or SSRIs) as fixed effects and participants as a random effect (with restricted maximum likelihood [REML] being applied). Effects of time, treatment, and time × treatment (interaction) on the dependent variable (MADRS, QIDS-CR, QIDS-SR, HAM-A, AIS, and SHAPS scores) were evaluated. Effect size was calculated as partial-eta squared for an interaction. Between-group comparisons (trazodone XR vs. SSRIs) were calculated for the estimated marginal means at each timepoint. Additional analysis was performed with the same method for all the outcomes with the duration of the previous psychiatric treatment included as a covariate in the model.

Internal consistency reliability was previously assessed in the pilot study (Siwek et al., 2023).

Proportions of treatment response and remission, as assessed with QIDS-CR, QIDS-SR, and MADRS, were compared between trazodone XR and SSRIs with the use of the χ2 test. Statistical significance was defined as a two-sided *p*-value of <0.05.

## 3 Results

### 3.1 Group characteristics

**FIGURE 1:**
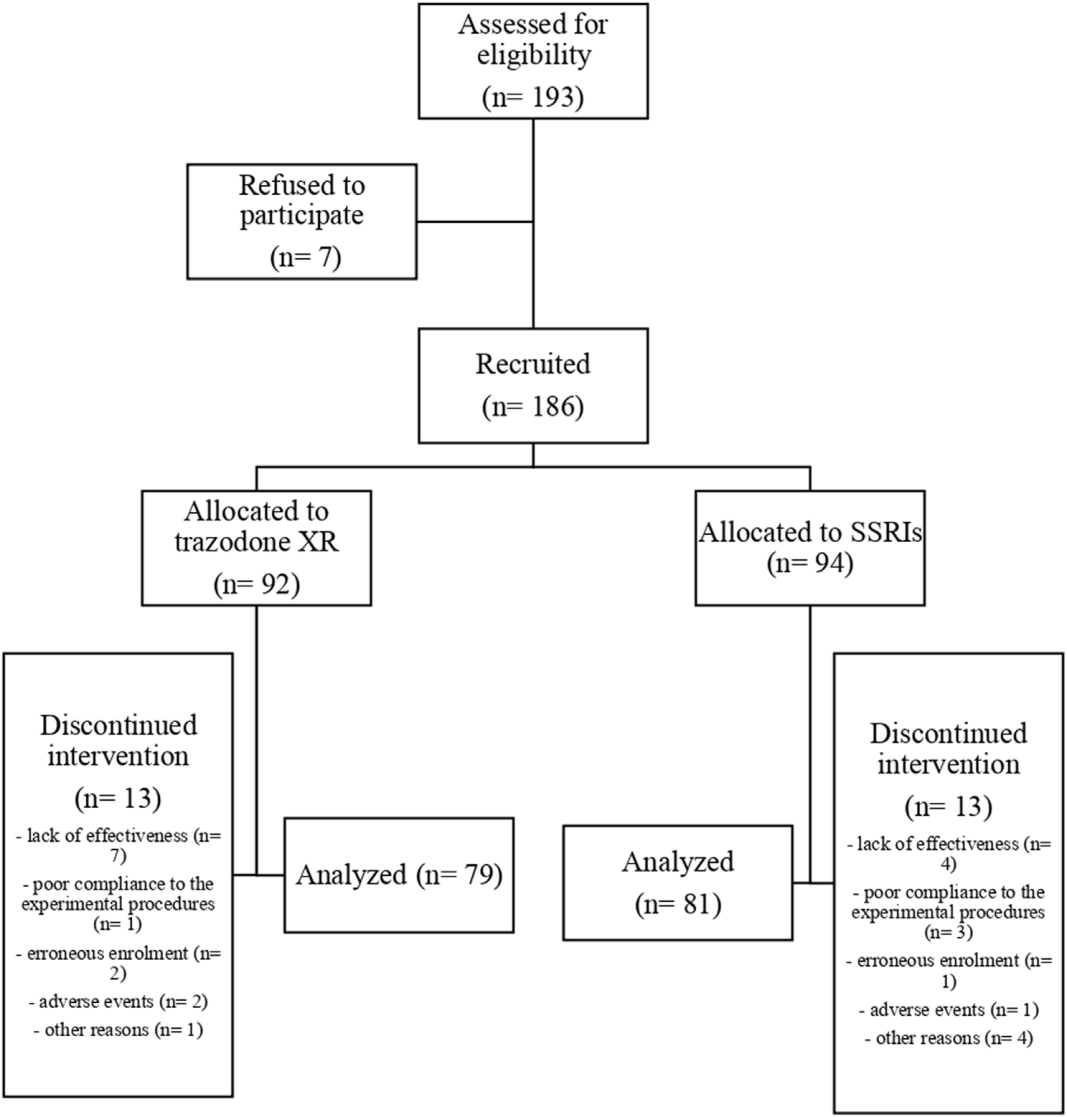
Flow chart of the study.

The flow chart of the study is presented in Figure 1. Comparisons of general group characteristics are presented in Table 1. The groups were comparable regarding sex, age, BMI, alcohol consumption, and presence of somatic comorbidities. The proportion of subjects who smoked was significantly higher in patients treated with trazodone XR vs. SSRIs. The duration of previous psychiatric treatment was longer in the subjects receiving trazodone XR vs. SSRIs. The severity of depression was comparable between the groups when assessed by MADRS and QIDS-SR; a trend for higher severity of depression was observed in the trazodone XR group vs. SSRIs group when evaluated by QIDS-CR (Table 1).

**TABLE 1:** Baseline group characteristics.

	SSRI (n = 81)	Trazodone XR (n = 79)	p-value
Sex (% female)	62.2%	50,65%	0.191^a^
Age (in years): mean (SD)	34.9 (12.9)	34.4 (12.0)	0.917^b^
BMI (in kilograms/m^2^): mean (SD)	23.9 (5.04)	24 (3.45)	0.907^b^
Duration of previous psychiatric treatment (in months)	21.3 (65.2)	32.6 (64.6)	0.046^b^
Alcohol consumption (% yes)	65.3%	71.8%	0.506^a^
Smoking (% yes)	7.2%	23.1%	0.018^a^
Somatic comorbidities (% yes)	28.1%	33.7%	0.582^a^
MADRS: mean (SD)	27.2 (7.41)	28.8 (7.26)	0.590^b^
QIDS-CR: mean (SD)	13.8 (4.07)	14.2 (4.38)	0.054^b^
QIDS-SR: mean (SD)	14.5 (4.59)	15.7 (4.72)	0.841^b^
SHAPS: mean (SD)	6.64 (4.48)	6.69 (4.29)	0.282^b^
HAMA: mean (SD)	20.6 (7.8)	21.5 (7.4)	0.452^b^
AIS: mean (SD)	10.4 (4.94)	13.7 (5.52)	0.161^b^

AIS, Athens Insomnia Scale; CGI-S, Clinical Global Impression Scale severity; HAM-A, Hamilton Anxiety Rating Scale; MADRS, Montgomery–Asberg Depression Rating Scale; QIDS-CR, Quick Inventory of Depressive Symptomatology—clinician-rated; QIDS-SR, Quick Inventory of Depressive Symptomatology—self-rated; SD, standard deviation; SHAPS, Snaith–Hamilton Pleasure Scale; XR, extended-release formulation.

^a^ Chi-squared test.

^b^ Independent-sample *t*-test.

### 3.2 Outcomes

The results of the MMRM models for each outcome measure are presented in Table 2. The effect of an interaction between time and the treatment type was statistically significant for scores in MADRS [F (4, 529.03) = 5.386, *p* < 0.001], QIDS-CR [F (4, 535.65) = 6.405, *p* < 0.001], QIDS-SR [F (4, 512.35) = 6.061, *p* < 0.001], HAM-A [F (4, 544.52) = 4.889, *p* < 0.001], AIS [F (4, 553.15) = 15.755, *p* < 0.001], and SHAPS [F (4, 550.86) = 2.495, *p* = 0.04]. Effect sizes for the time × treatment interaction (measured by the partial-eta squared, η2) were moderate for AIS (η2 = 0.1) and small for QIDS-CR (η2 = 0.05), QIDS-SR (η2 = 0.05), MADRS (η2 = 0.04), HAMA (η2 = 0.03), and SHAPS (η2 = 0.02) (Table 2).

**TABLE 2:** Results of mixed-effect model–significance levels and effect sizes (partial-eta squared) for all outcomes.

	Time effect, p	Treatment effect, p	Time × treatment effect, p	Partial-eta squared for interaction (with 95% CI)
MADRS	<0.001	0.308	<0.001	0.04 (0.01–0.07)
QIDS-CR	<0.001	0.019	<0.001	0.05 (0.01–0.08)
QIDS-SR	<0.001	0.566	<0.001	0.05 (0.01–0.08)
HAM-A	<0.001	0.299	<0.001	0.03 (0.01–0.06)
AIS	<0.001	0.186	<0.001	0.1 (0.06–0.15)
SHAPS	<0.001	0.164	0.04	0.02 (0.00–0.04)
BMI	0.869	0.856	0.821	<0.001 (0.00–0.01)

AIS, Athens Insomnia Scale; HAM-A, Hamilton Anxiety Rating Scale; MADRS, Montgomery–Asberg Depression Rating Scale; QIDS-CR, Quick Inventory of Depressive Symptomatology—clinician-rated; QIDS-SR, Quick Inventory of Depressive Symptomatology—self-rated; SHAPS, Snaith–Hamilton Pleasure Scale.

The estimated marginal means for each outcome measure are displayed in Table 3, separately at each timepoint (baseline, 2, 4, 8, and 12 weeks) with appropriate *p*-values for comparisons between SSRIs and trazodone. Statistically significant differences between SSRIs and trazodone XR in favor of trazodone were noted in MADRS at 8 weeks (11.2 vs. 8.03, *p* = 0.039) and at 12 weeks (11.06 vs. 5.99, *p* = 0.001); QIDS-CR (6.71 vs. 3.21, *p* < 0.001), QIDS-SR (7.66 vs. 5, *p* = 0.003), and SHAPS (4.42 vs. 2.56, *p* = 0.011) at 12 weeks; HAM-A at 8 weeks (6.98 vs. 4.27, *p* = 0.03) and at 12 weeks (7.46 vs. 3.7, *p* = 0.003); and AIS at baseline (10.42 vs. 13.73, *p* < 0.001), at 4 weeks (7.68 vs. 5.72, *p* = 0.014), at 8 weeks (6.11 vs. 4.39, *p* = 0.034), and at 12 weeks (7.04 vs. 3.88, *p* < 0.001) (Table 3).

**TABLE 3:** Between-group comparisons for each timepoint.

	Baseline emmean (95% CI)			2 weeks emmean (95% CI)			4 weeks emmean (95% CI)			8 weeks emmean (95% CI)			12 weeks emmean (95% CI)		
	SSRI	T-XR	p-value	SSRI	T-XR	p-value	SSRI	T-XR	p-value	SSRI	T-XR	p-value	SSRI	T-XR	p-value
MADRS	27.29 (25.3–29.3)	28.74 (26.69–30.8)	0.318	19.19 (17.15–21.2)	20.6 (17.97–22.2)	0.556	12.77 (10.73–14.8)	13.41 (11.28–15.5)	0.67	11.22 (9.11–13.3)	8.03 (5.86–10.2)	0.039	11.06 (8.94–13.2)	5.99 (3.78–8.2)	0.001
QIDS-CR	13.69 (12.54–14.83)	14.2 (13.02–15.39)	0.537	10.16 (8.98–11.33)	10.1 (8.91–11.29)	0.946	7.31 (6.14–8.48)	5.91 (4.77–7.16)	0.116	6.57 (5.36–7.77)	3.79 (2.55–5.02)	0.002	6.79 (5.58–8.00)	3.21 (1.95–4.47)	<0.001
QIDS-SR	14.26 (13.08–15.43)	15.6 (14.3–16.9)	0.133	10.86 (9.68–12.04)	12.17 (10.95–13.39)	0.13	8.69 (7.52–9.87)	8.07 (6.83–9.32)	0.476	7.4 (6.21–8.59)	6.21 (4.94–7.48)	0.178	7.66 (6.45–8.88)	5 (3.71–6.29)	0.003
HAM-A	20.51 (18.87–22.15)	21.34 (19.59–23.09)	0.499	12.35 (10.68–14.01)	13.18 (11.46–14.9)	0.495	7.45 (5.77–9.13)	7.66 (5.92–9.39)	0.865	6.98 (5.3–8.67)	4.27 (2.5–6.04)	0.03	7.46 (5.57–9.18)	3.7 (1.88–5.51)	0.003
AIS	10.42 (9.37–11.47)	13.73 (12.64–14.81)	<0.001	9.07 (7.98–10.15)	8.9 (7.82–9.98)	0.826	7.68 (6.61–8.76)	5.73 (4.6–6.86)	0.014	6.11 (5.03–7.19)	4.39 (3.23–5.55)	0.034	7.04 (5.92–8.16)	3.88 (2.7–5.05)	<0.001
SHAPS	6.62 (5.69–7.55)	6.65 (5.67–7.63)	0.969	5.29 (4.33–6.25)	5.65 (4.69–6.62)	0.601	4.42 (3.44–5.4)	3.4 (2.38–4.41)	0.154	3.67 (2.71–4.64)	2.69 (1.66–3.72)	0.172	4.43 (3.43–5.43)	2.56 (1.51–3.6)	0.011
BMI	23.9 (22.9–24.9)	24 (22.9–25)	0.907	23.8 (22.8–24.8)	23.9 (22.9–24.9)	0.931	23.8 (22.8–24.8)	24 (22.9–25)	0.807	23.8 (22.8–24.8)	24 (22.9–25)	0.842	23.8 (22.8–24.8)	23.9 (22.9–25)	0.865

Values are presented as estimated marginal means with 95% confidence intervals. Emmean, estimated marginal mean. AIS, Athens Insomnia Scale; HAM-A, Hamilton Anxiety Rating Scale; MADRS, Montgomery–Asberg Depression Rating Scale; QIDS-CR, Quick Inventory of Depressive Symptomatology—clinician-rated; QIDS-SR, Quick Inventory of Depressive Symptomatology—self-rated; SHAPS, Snaith–Hamilton Pleasure Scale, T-XR, trazodone extended-release formulation.

The results of the MMRM models for each assessed outcome, with the duration of previous psychiatric treatment as a covariate, are depicted in Table 4. There was a statistically significant effect of the interaction between time and treatment type for the scores in MADRS [F (4, 471.9] = 5.79, *p* < 0.001), QIDS-CR [F (4, 479.9) = 14.02), *p* < 0.001], QIDS-SR [F (4, 453.4) = 5.07), *p* < 0.001], HAM-A [F (4, 485.4) = 4.8), *p* < 0.001], and AIS [F (4, 490.9) = 14.01), *p* < 0.001]. The effect of the interaction between time and the treatment type for the scores in the SHAPS showed a trend which did not reach the level of statistical significance [F (4, 491.9) = 2.24, *p* = 0.06]. The effect sizes for the time–treatment interaction (measured by the partial-eta square, η2) were moderate for the AIS (η2 = 0.1) scale and small for the MADRS (η2 = 0.04), QIDS-CR (η2 = 0.05), QIDS-SR (η2 = 0.05), and HAM-A (η2 = 0.03) scales (Table 4).

**TABLE 4:** Results of the mixed-effect model, with the duration of previous psychiatric treatment as a covariate, showing the significance levels and effect sizes (partial-eta squared) for all outcomes.

	Treatment effect, p	Time effect, p	Time × treatment effect, p	Partial-eta squared for interaction (with 95% CI)
MADRS	0.590	<0.001	<0.001	0.04 (0.01–0.07)
QIDS-CR	0.054	<0.001	<0.001	0.05 (0.01–0.08)
QIDS-SR	0.841	<0.001	<0.001	0.05 (0.01–0.08)
HAM-A	0.452	<0.001	<0.001	0.03 (0.01–0.07)
AIS	0.161	<0.001	<0.001	0.10 (0.06–0.15)
SHAPS	0.282	<0.001	0.064	0.02 (0.00–0.04)

MADRS, Montgomery–Asberg Depression Rating Scale; HAM-A, Hamilton Anxiety Rating Scale; QIDS-CR, Quick Inventory of Depressive Symptomatology—clinician-rated; QIDS-SR, Quick Inventory of Depressive Symptomatology, self-rated; SHAPS, Snaith–Hamilton Pleasure Scale; AIS, Athens Insomnia Scale.

**FIGURE 2:**
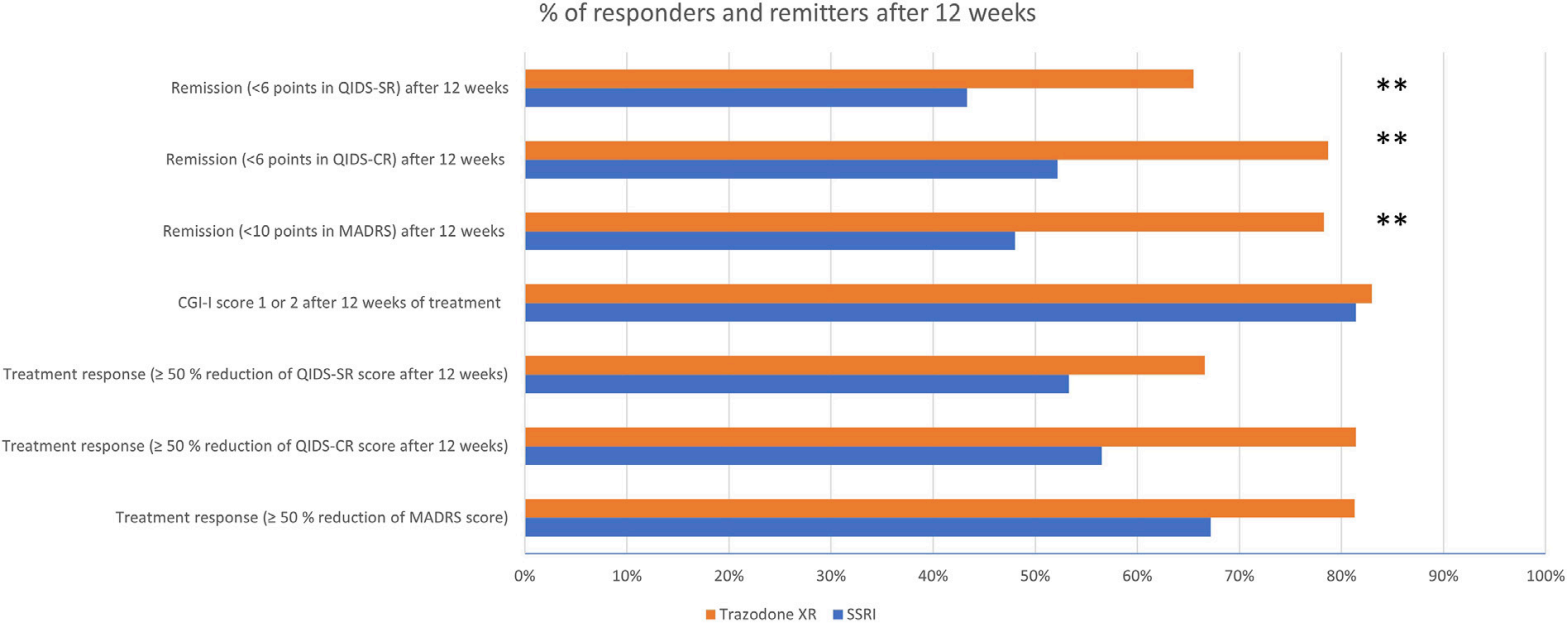
Proportions of patients achieving treatment response and remission in in SSRIs vs. trazodone XR groups.

Table 5 and Figure 2 show the comparison of proportions of patients achieving treatment response and remission in SSRIs vs trazodone XR groups, as assessed after 12 weeks. As measured by QIDS-CR, the proportion of participants achieving treatment response was higher in the trazodone XR vs. SSRIs group. As assessed by MADRS, QIDS-SR, and CGI-I, no statistically significant differences in the proportion of individuals achieving treatment response between the SSRIs and trazodone XR groups were noted. Total scores of MADRS, QIDS-CR, and QIDS-SR indicated that the proportion of participants achieving remission was higher in the trazodone XR vs. SSRI group (Table 5; Figure 2).

**TABLE 5:** Comparison of frequencies of therapeutic response, remission (measured by QIDS-CR, QIDS-SR, and MADRS scores), and clinical improvement (measured by CGI-I) between patients treated with SSRI and trazodone XR after 12 weeks of treatment.

	SSRI	Trazodone XR	p-value
Treatment response (≥50% reduction of the MADRS score after 12 weeks), % of patients	67.2	81.3	0.082
Treatment response (≥50% reduction of the QIDS-CR score after 12 weeks), % of patients	56.5	81.4	0.006
Treatment response (≥50% reduction of the QIDS-SR score after 12 weeks), % of patients	53,3	66.6	0.24
CGI-I score 1 or 2 after 12 weeks of treatment, % of patients	81.4	83	0.113
Remission (<10 points in MADRS) after 12 weeks, % of patients	48	78.3	0.012
Remission (<6 points in QIDS-CR) after 12 weeks, % of patients	52.2	78.7	0.003
Remission (<6 points in QIDS-SR) after 12 weeks, % of patients	43.3	65.5	0.021

CGI-I, Clinical Global Impression Scale—improvement; MADRS, Montgomery–Asberg Depression Rating Scale; QIDS-CR, Quick Inventory of Depressive Symptomatology—clinician-rated; QIDS-SR, Quick Inventory of Depressive Symptomatology—self-rated.

## 4 Discussion

The results showed that both SSRIs and trazodone XR were effective in reducing the overall symptoms of depression, anxiety, insomnia, and anhedonia. No significant effects of the treatment on BMI were noted in neither of the treatment groups. The discontinuation levels were 16.46% in the trazodone XR group and 16.05% in the SSRI group.

Baseline comparisons showed that both treatment arms were similar regarding the severity of anxiety, insomnia, and anhedonia. The baseline scores of depression varied depending on the tool used for evaluation; two scales indicated that levels of depression were comparable in both groups, while one showed a trend for higher levels of depression in the trazodone XR group vs. SSRIs group which did not reach statistical significance. There is a plethora of depression clinical presentations which vary greatly, and the tools used to measure the severity of depression are considerably dissimilar in the range of assessed symptoms. In addition, it is known that clinician and self-rated tools are not interchangeable in evaluating depressive symptoms, even despite the same content, and using either clinician or self-assessed scales might not provide a thorough evaluation of the clinical picture. It is, therefore, highly advisable to use more than one tool to measure affective symptoms and include both patient- and clinician-rated scales in order to increase the credibility and replicability of results (Uher et al., 2012; Fried, 2017; Chrobak et al., 2018).

Moreover, the MMRM analysis indicated that trazodone XR was more effective than SSRIs in reducing the levels of depression measured by MADRS, QIDS-SR, and QIDS-CR; anxiety assessed by HAM-A; and insomnia evaluated by AIS; these results remained significant after controlling for the duration of previous psychiatric treatment as a covariate. The initial MMRM analysis suggested that trazodone XR was also more effective in improving anhedonia assessed by SHAPS, but after controlling for the duration of previous psychiatric treatment, this trend did not reach statistical significance. Relatively high treatment response and remission rates were noted in both study arms. The estimated marginal means showed that, compared to SSRIs, the benefits of trazodone XR treatment were noticeable after 4 weeks, regarding insomnia, and 8 weeks, regarding the severity of depression and anxiety.

The proportion of treatment-responsive subjects in the trazodone XR group compared to the SSRI group was higher when assessed by QIDS-CR (81.4% vs. 56.5%), or comparable when evaluated with MADRS (81.3% vs. 67.2%), QIDS-SR (66.6% vs. 53.3%), or CGI-I (83% vs. 81.4%). The proportion of patients achieving remission was statistically significantly higher in the trazodone XR arm vs. SSRI arm, as assessed with MADRS (78.3% vs. 48%), QIDS-CR (78.7% vs. 52.2%), and QIDS-SR (65.5% vs. 43.3%). In contrast, in the largest to date “real-world” study on the effectiveness of antidepressant treatment (citalopram), STAR*D treatment response was noted in 47% and remission in 28%–33% of participants. Several factors might have influenced our results (Trivedi et al., 2006). First, this was a single-centered study performed in a university psychiatric clinic, while STAR*D was a multi-centered study performed in both psychiatric and primary care settings. Second, patients enrolled in the STAR*D study had a mean duration of illness of 15 years, while in our study, the mean duration of illness was 21.3 months in the SSRI group and 32.6 in the trazodone XR group. Third, the inclusion criteria in our trial were stricter than in STAR*D, i.e., we did not include subjects with persistent mood disorder, while in the former study, these patients constituted approximately 25% of the study sample; our observation excluded individuals with substance abuse (other than caffeine of nicotine), while in the STAR*D study, participants with alcohol or drug dependence accounted for nearly 20% of the sample. These factors could explain the differences in the rates of treatment response and remission in our observation, given that a longer duration of depressive symptoms and/or substance abuse are liked to lower treatment effectiveness of antidepressant therapy (Ghio et al., 2014; Agabio et al., 2018). Our results are consistent with the previous findings, suggesting that trazodone (CR or IR) is no less effective than fluoxetine (Falk et al., 1989; Beasley et al., 1991), paroxetine (Kasper et al., 2005), or sertraline (Munizza et al., 2006) in MDD and more beneficial in improving insomnia (Papakostas and Fava, 2007). On the other hand, a more recent RCT indicated that venlafaxine XR was more effective than trazodone XR in achieving remission of depressive symptoms, while the results regarding reduction of depression severity and achievement of treatment response were inconsistent, indicating comparable or superior effectiveness of venlafaxine XR vs. trazodone XR. In congruence with this study, we observed that the reduction of depressive symptoms occurs earlier in the course of treatment in trazodone XR vs. SSRI-treated subjects (Fagiolini et al., 2020). Noteworthy, all the previous studies comparing trazodone to SSRIs were double-blind clinical trials, and all, but one, used only clinician-rated tools to assess treatment outcomes, especially the Hamilton Depression Rating Scale, MADRS, and/or CGI. None administered specific tools to evaluate the changes in insomnia or anhedonia. Adding to the pilot results of this study (Siwek et al., 2023), we noted that compared to SSRIs, trazodone XR was more effective in improving not only depression and insomnia but also anxiety. Although the pilot showed no significant differences in the levels of treatment response and remission, the analysis of the complete sample indicated that trazodone XR was more effective than SSRIs in achieving the remission of depressive symptoms. The results were inconsistent regarding the levels of treatment response as one of the scales suggested higher effectiveness of trazodone XR vs. SSRIs (QIDS-CR), while the three others did not (MADRS, QIDS-SR, and CGI-I).

The novelty of our work lies in several methodological attributes: 1) the use of three tools assessing depression severity, both clinician- and patient-rated, which provide a more thorough assessment of trazodone XR effectiveness in reducing overall depression severity; 2) the use of specific scales to measure the severity of different symptom dimensions such as AIS, HAM-A, and SHAPS, and which offer more precise knowledge on the effectiveness of trazodone XR in particular symptomatic dimensions, and 3) the choice of naturalistic observation design, which is more easily translated to the “real-life” settings than RCT. Indeed, the focused assessment of anhedonia is an important advancement in the evaluation of depression clinical presentation as it is known that improvement in positive affect and hedonic tone is more relevant to functional remission than changes in negative affect, which are the focus of the depression assessment tools (HAM-D) (Demyttenaere et al., 2021). Moreover, the thorough measurement of insomnia is of significant value as it is one of the most common reasons for treatment discontinuation (Badamasi et al., 2019) and resistance (Wade, 2006).

Our work has several limitations. The naturalistic, open-label design of the study and lack of randomization may have contributed to the dissimilarity of treatment arms. Nonetheless, this did not obstruct the potential of this work to show the non-inferiority of trazodone XR vs. SSRIs (as the trazodone XR arm presented a longer previous psychiatric treatment duration, which could potentially lower the effectiveness of the drug). Other potentially confounding factors were the single-center design, differences in antidepressant doses, and inclusion of various SSRIs in the same group. Because of this, the results require replications in studies with more robust methodology. However, this does not hamper the advances provided in our work, which are due to a more thorough assessment of the depressive symptomatology. Given the non-inferiority design, we might only dispute on the comparableness of trazodone XR vs. SSRIs, but the speculations on its superiority to SSRIs remain to be verified in future trials.

## 5 Conclusion

In summary, our results demonstrate that trazodone XR is a valuable MDD treatment option as SSRIs. The potential superiority of trazodone XR vs. SSRIs in improving overall depression, anhedonia, and insomnia, and achieving remission should be further evaluated in future studies.

## Data Availability

The raw data supporting the conclusion of this article will be made available by the authors, without undue reservation.
